# Synaptic and Endosomal Localization of Active γ-Secretase in Rat Brain

**DOI:** 10.1371/journal.pone.0008948

**Published:** 2010-01-28

**Authors:** Susanne Frykman, Ji-Yeun Hur, Jenny Frånberg, Mikio Aoki, Bengt Winblad, Jarmila Nahalkova, Homira Behbahani, Lars O. Tjernberg

**Affiliations:** 1 Department of Neurobiology, Care Sciences and Society (NVS), Karolinska Institutet Dainippon Sumitomo Pharma Alzheimer Center (KASPAC), Novum, Huddinge, Sweden; 2 Department of Neurobiology, Care Sciences and Society (NVS), Karolinska Institutet Alzheimer's Disease Research Center (KI-ADRC), Karolinska Institutet, Novum, Stockholm, Sweden; Universidade Federal do Rio de Janeiro (UFRJ), Brazil

## Abstract

**Background:**

A key player in the development of Alzheimer's disease (AD) is the γ-secretase complex consisting of at least four components: presenilin, nicastrin, Aph-1 and Pen-2. γ-Secretase is crucial for the generation of the neurotoxic amyloid β-peptide (Aβ) but also takes part in the processing of many other substrates. In cell lines, active γ-secretase has been found to localize primarily to the Golgi apparatus, endosomes and plasma membranes. However, no thorough studies have been performed to show the subcellular localization of the active γ-secretase in the affected organ of AD, namely the brain.

**Principal Findings:**

We show by subcellular fractionation of rat brain that high γ-secretase activity, as assessed by production of Aβ40, is present in an endosome- and plasma membrane-enriched fraction of an iodixanol gradient. We also prepared crude synaptic vesicles as well as synaptic membranes and both fractions showed high Aβ40 production and contained high amounts of the γ-secretase components. Further purification of the synaptic vesicles verified the presence of the γ-secretase components in these compartments. The localization of an active γ-secretase in synapses and endosomes was confirmed in rat brain sections and neuronal cultures by using a biotinylated γ-secretase inhibitor together with confocal microscopy.

**Significance:**

The information about the subcellular localization of γ-secretase in brain is important for the understanding of the molecular mechanisms of AD. Furthermore, the identified fractions can be used as sources for highly active γ-secretase.

## Introduction

Alzheimer's disease (AD) is the most common neurodegenerative disease and the prevalence is increasing with the longer life span of the human population. The disease is characterized by memory loss and other cognitive deficits as well as the pathological hallmarks amyloid plaques and neurofibrillar tangles. The amyloid plaques consist of fibrils of amyloid β-peptide (Aβ) that also can form soluble, neurotoxic oligomers. Aβ is produced from the amyloid precursor protein (APP) through two sequential cleavages performed by β- and γ-secretase. In addition, APP can also be processed by a non-amyloidogenic pathway by α-secretase and γ-secretase which results in the non-toxic P3 peptide. In both cases, the APP intracellular domain (AICD) is released into the cytosol upon γ-secretase cleavage. γ-Secretase is a transmembrane complex consisting of at least four proteins; presenilin (PS), nicastrin, anterior pharynx defective-1 (Aph-1) and presenilin enhancer-2 (Pen-2). During maturation of the complex, presenilin is endoproteolytically cleaved to form an N-terminal and a C-terminal fragment (NTF and CTF respectively). There are two isoforms of presenilin (PS1 and PS2) and three isoforms of Aph-1 (Aph-1aL, Aph-1aS and Aph1b) in humans. In addition to APP, γ-secretase cleaves more than 60 other substrates which results in a high risk of side-effects when targeting γ-secretase as a therapeutic strategy [1].

To fully understand the mechanism of how Aβ causes Alzheimer's disease it is important to localize the subcellular compartments in which Aβ is produced. The subcellular localization could also play a role in substrate selectivity and detailed knowledge on the processing would be helpful in designing therapeutic compounds that specifically inhibits Aβ production. Several studies have explored the subcellular localization of γ-secretase in different cell lines and come to the conclusion that the mature γ-secretase complex is mainly present in the late secretory and/or endosomal pathways [2], [3], [4], [5] and that the assembly of the complex appears to be initiated in the endoplasmatic reticulum (ER) [6]. In addition, lysosomal [7] and autophagosomal localization of the complex has been reported [8] and a small proportion of the γ-secretase activity can be found at the cell surface [9] and in the mitochondria [10]. To our knowledge, however, no extensive study has been performed to elucidate the subcellular localization of an active γ-secretase in brain. Neurons differ considerably from other cells and it is plausible that also the subcellular localization of proteins is different in these cells. For example, it was shown that Aβ was mainly found in intracellular compartments in neurons, whereas the majority of Aβ was secreted in COS-7 cells [11]. In addition, in some of the studies the γ-secretase components were overexpressed which can affect the subcellular localization. Although the subcellular localization of Aβ in brain tissue has been found to be mainly endosomal [12], [13], it cannot be ruled out that this pool of Aβ was produced in another subcellular compartment and/or endocytosed from the extracellular space. The presenilins were the first γ-secretase components to be discovered and their subcellular localization has been determined in brain [14], [15], [16], [17], [18], [19]. Interestingly, besides the localization to different cell body compartments, presenilin was also found in synaptic compartments [14], [15], [17], [18]. Synaptic degeneration is one of the first hallmarks of Alzheimer's disease and thus the potential production of the toxic Aβ at this site is likely to be of importance for the disease progression. Naturally, synaptic compartments can only be studied in brain or cultured neurons and are thus not included in the earlier studies where the subcellular localization of the active γ-secretase complex was determined using non-neuronal cells.

We have performed an extensive subcellular fractionation to elucidate the subcellular localization of active γ-secretase in rat brain. In addition to monitoring all the γ-secretase components in the subcellular fractions, we have also studied the γ-secretase activity, measuring AICD and Aβ40 production in these fractions. In addition we used confocal microscopy and a biotinylated γ-secretase active site inhibitor to label γ-secretase in rat brain sections or mouse primary neurons. Both methods indicate an endosomal and synaptic localization of active γ-secretase, suggesting that these compartments to a large degree contribute to the γ-secretase activity in neurons.

## Materials and Methods

### Antibodies

The following antibodies were used in this study: nicastrin, (MAB5556, Chemicon); PS1-CTF (MAB5232, Chemicon); PS2-CTF (Calbiochem); Aph-1aL (BioSite); UD1 raised against Pen-2 (a gift from Dr. Jan Näslund, Karolinska Institutet, Sweden); C1/6.1, raised against the C-terminus of APP (a gift from Dr. Paul M. Mathews, Nathan Kline Institute, NY, USA); N-cadherin (BD Biosciences); syntaxin 13 (Stressgen); γ-Adaptin (BD Biosciences); GM130 (clone: 35, BD Biosciences); ERGIC-53 (Affinity Bioreagents) and KDEL (BioSite); PSD-95 (K28/43, Upstate Cell Signaling Solutions); Synaptohysin (Chemicon), Rab5 (ab18211, Abcam), synapsin1 (Invitrogen, ZYMED Laboratories).

### Animals

Male Sprague Dawley rats and pregnant C57BL/6 mice were obtained from Scanbur AB or Taconic. The rats used in this study were treated according to the Karolinska Institutet as well as national guidelines and the study was approved by the Animal research ethical committee of southern Stockholm. No experiments were performed on live animals.

### Iodixanol Gradient Centrifugation

Male Sprague Dawley rats were sacrificed by carbon dioxide and the cerebellum, white matter and blood vessels were removed. One half of a brain was homogenized in 1.5 ml Buffer I (130 mM KCl, 25 mM Tris-HCl, pH 7.4, 1 mM EGTA) including Complete protease inhibitor cocktail (Roche). The homogenates were centrifuged at 1 000×g for 10 min to remove nuclei and at 10 000×g for 15 min to remove mitochondria and synaptosomes. The 10 000×g supernatant was layered on an iodixanol gradient consisting of 1 ml each of 30, 25, 20, 15, 12.5, 10, 7.5, 5 and 2.5% (w/v) iodixanol (Sigma) in Buffer I and centrifuged at 126 000×g for 40 min. 1 ml fractions was collected from the bottom of the tube, diluted 4 times with Buffer I and centrifuged at 126 000×g for 40 min. The pellets were resuspended in Buffer H (150 mM NaCl, 20 mM Hepes-KOH, pH 7.0, 5 mM EDTA and protease inhibitor cocktail) with 0.4% CHAPSO.

### Preparation of Synaptic Membranes and Vesicles

Synaptic membranes and vesicles were prepared according to Cohen *et al* [20]. Briefly, rat brains were obtained as above and homogenized in Buffer A (0.32 M sucrose, 1 mM NaHCO_3_, 1 mM MgCl_2_, 0.5 mM CaCl_2_). The P2 (17 300×g) pellet was resuspended in Buffer B (1 mM NaHCO_3_, 0.32 M sucrose) and layered on a sucrose gradient to purify synaptosomes. The synaptosomes were lysed in 6 mM Tris-HCl pH 8.1 and the lysate was centrifuged at 48 250×g to separate membranes (LP1) from synaptic vesicles. Synaptic membranes were further purified from LP1 on a second sucrose gradient to remove synaptic mitochondria, whereas the synaptic vesicles were pelleted at 100 000×g for 2 h. The 17 300×g supernatant was centrifuged at 100 000×g for 1 h to obtain a reference pellet (P3). In addition, highly pure synaptic vesicles were kindly provided by Dr. Matthew Holt, Goettingen, Germany. The vesicles were prepared according to Hüttner et al [21], including controlled pored glass chromatography (CPG). Protein concentrations were determined by BCA™ protein assay kit (Pierce).

### Gel Electrophoresis and Immunoblotting

The fractions were loaded onto 4–12% polyacrylamide bis-tris gels or 10–20% polyacrylamide tricine gels and transferred to PVDF membranes which were incubated with primary antibodies followed by HRP-coupled secondary antibodies (GE Healthcare) and SuperSignal substrate (Pierce). For quantification of PSD-95 and synaptophysin, 0.02, 0.1, 0.5 and 2,5 µg of protein of the homogenate, synaptic membranes and synaptic vesicles were loaded on the gel.The signals were quantified either by using a CCD-camera (Fuji LAS3000) (synaptic fractions) or by exposure to film which was scanned on a flat-bed scanner and the image was quantified by a Fluor-S Max CCD camera (BioRad) (iodixanol fractions). In the latter case, precautions were taken not to use overexposed films.

### Electron Microscopy

The fractions were fixed in 2% glutaraldehyde in 0.1 M sodiumcacodylate, pH 7.4, 0.1 M sucrose, 3 mM CaCl_2_, at 4°C over night, and centrifuged to a pellet. The pellet was rinsed in 0.15 M sodiumcacodylate buffer containing 3 mM CaCl_2_, pH 7.4 followed by postfixation in 2% osmium tetroxide in 0.07 M sodiumcacodylate buffer containing 1.5 mM CaCl_2_, pH 7.4 at 4°C for 2 hours, dehydrated in ethanol followed by acetone and embedded in LX-112 (Ladd). Sections were contrasted with uranyl acetate followed by lead citrate and examined in a Leo 906 transmission electron microscope at 80 kV. Digital images were taken by using a Morada digital camera (Soft Imaging System, GmbH).

### γ-Secretase Activity Assay

The fractions were incubated in buffer H (20 mM Hepes, 150 mM NaCl, 5 mM EDTA, pH 7.0) including 0.4% CHAPSO and a protease inhibitor cocktail (Roche) at 37°C for 16 h with or without 1 µM of the γ-secretase inhibitor L-685,458 (Bachem). 0.4% CHAPSO was used since we have earlier found this to be the optimal detergent concentration [22]. For iodixanol fractions one third of each fraction was used for each sample. For synaptic fractions 100 µg of sample was used. For some samples, 20 ng of C99-FLAG (a kind gift from Dr. Takeshi Nishimura, Dainippon Sumitomo Pharma) solubilized in tri-fluoro-ethanol (TFE), was added prior to incubation. Production of AICD was analyzed by immunoblotting and production of Aβ40 was analyzed by a commercial ELISA system (Wako chemicals). Prior to ELISA, the reactions were stopped by the addition of RIPA buffer, heated to 95°C for 5 min and centrifuged at 16 000×g for 5 min. The supernatants were subjected to ELISA according to the manufacturer's protocol. Production of Aβ40 was calculated as Aβ40 levels without L-685,458 minus Aβ40 levels with added L-685,458. For AICD degradation, 0.2 ng of synthetic AICD (Calbiochem) and 1 µM of L-685,458 were added to the fractions prior to incubation. Synthetic AICD without any fraction was used as a control.

### Statistical Analysis

The Aβ40 production in the different synaptic fractions was compared with the production in homogenates using unpaired t-test.

### Labeling of Rat Brain Sections with a Biotinylated γ-Secretase Inhibitor

Cryopreserved rat brain sections from frontal cortex embedded in OCT-compound (Tissue-TEK), were cut in 12 µm thick sections, mounted on Hypertema Teflon-coated (HTC) glass slides (Novakemi) and air dried. Primary cortical neuron cultures were established from cortices dissected from E17 mice (C57BL/6) and prepared as previously described [23]. The rat brain tissues or mouse primary neurons were fixed in buffered 4% (v/v) formaldehyde for 5 min at room temperature (RT). The tissues or cells were permeabilized with 0.2% Triton X-100 for 20 min following blocking with a Avidin/Biotin blocking Kit (Vector Laboratories, Inc.) for 15 min each. Blocking was done with DAKO protein block serum-free for 30 min at RT after washing with phosphate buffered saline (PBS). The tissues and cells were preincubated with 50 µM L-685,458 for 5 min at RT, followed by incubation with 500 nM GCB (γ-secretase inhibitor with cleavable biotin group, [24]) for 10 min, RT. Subsequently, the tissues or cells were incubated with Streptavidin-Alexa 488 (Invitrogen, Molecular Probes Inc.) for 30 min at 37°C. Later, incubations with primary antibodies were performed at 37°C, 1 h for brain tissues and at 4°C, over night for primary neurons. After washing with PBS, the sections or cells were incubated with secondary antibodies; anti-rabbit or anti-mouse AlexaFluor 546-conjugated IgG (Invitrogen, Molecular Probes Inc.) diluted in 2% normal goat serum for 15 min at 37°C. To reduce the background of staining in tissues, we used autofluorescence eliminator reagent (Chemicon International). All samples were visualized using an inverted laser scanning microscope (LSM 510 META; Zeiss).

## Results

### γ-Secretase Co-Fractionates with Endosomes and the Plasma Membrane

We have previously shown that the γ-secretase activity is highly enriched in a 100 000×g pellet prepared from rat brain containing Golgi, ER and endosomes [22]. In order to further determine the subcellular compartment with the highest γ-secretase activity we performed a density gradient centrifugation. To improve the separation of organelles, we used a 10 000×g supernatant instead of the 100 000×g pellet as starting material and we noted that iodixanol was superior to sucrose in this matter (data not shown).

**Figure 1:**
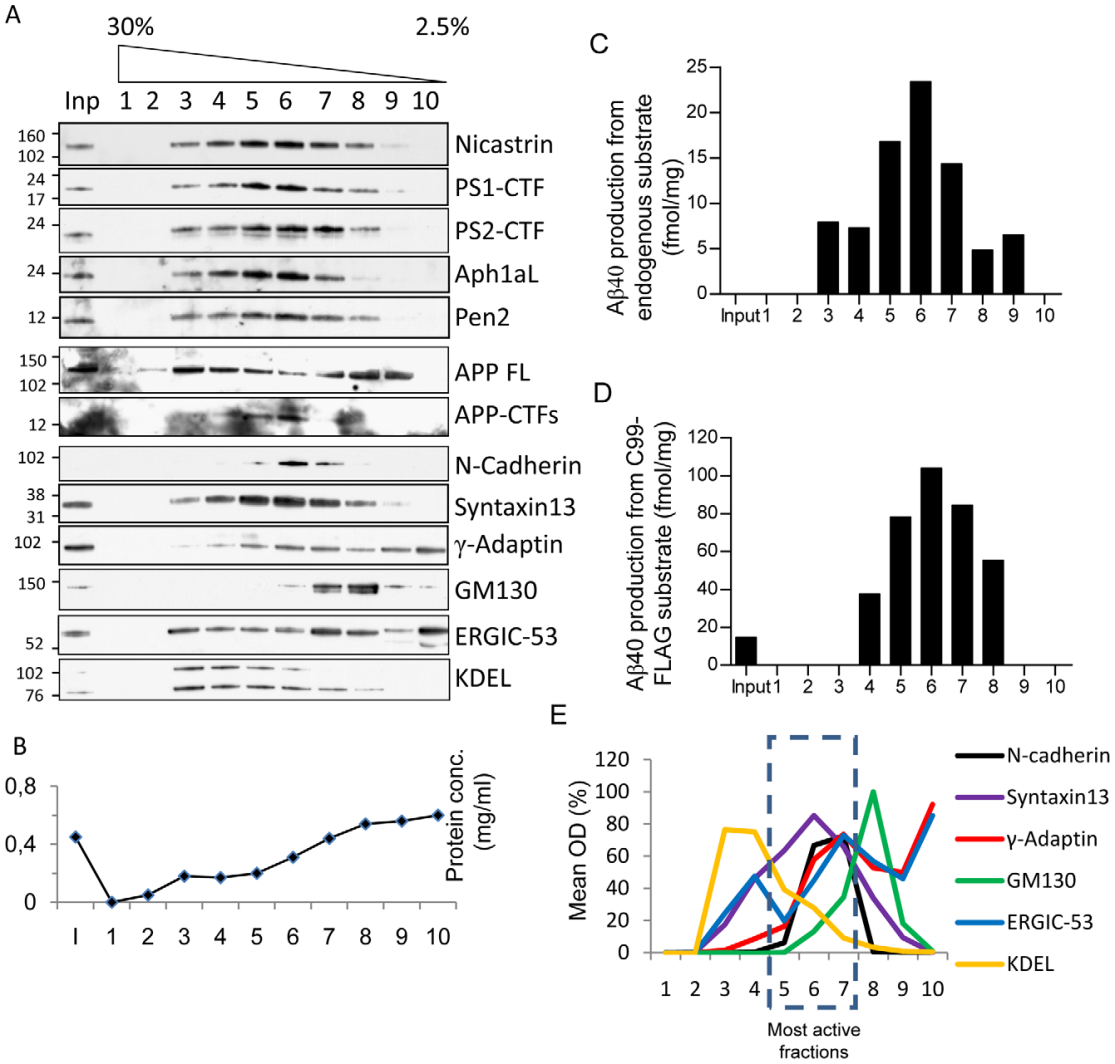
γ-Secretase components and activity in a discontinuous iodixanol gradient. Postmitochondrial supernatant from rat brain was layered on a 2.5–30% iodixanol gradient and fractions were collected from the bottom of the tube. A) Western blots showing the γ-secretase components, the substrate APP and the subcellular markers N-cadherin (plasma membrane), syntaxin 13 (early endosomes), γ-adaptin (trans-Golgi network), GM130 (cis-Golgi), ERGIC53 (ER-Golgi intermediate compartment) and KDEL (endoplasmatic reticulum). Each marker was analyzed in 3–4 gradients. B) Protein concentration in the fractions. Note that the protein concentration does not peak in the same fraction as the γ-secretase components or activity. The line is added just to guide the eye. C) Aβ40 production from endogenous substrate. The fractions were incubated over night at 37°C with or without L-685,458 and the Aβ40 concentration was measured by ELISA. Production was calculated as concentration without inhibitor minus concentration with inhibitor. D) Aβ40 production with added exogenous substrate (C99-FLAG). The fractions were incubated as above but with the addition of C99-FLAG. Due to slight discrepancies in the peak fraction between experiments which resulted in large standard deviations for each fraction, we have chosen to show a representative experiment rather than the mean value. The peak fraction was, however, always fraction 5, 6 or 7 and the enrichment in the peak fraction was always at least three-fold. Each experiment was repeated 5 times. E) Quantification of the subcellular markers in the different fractions. The fractions with highest γ-secretase activity are indicated by the dotted box. Mean values (% of optical density (OD) in the fraction with the highest density for each marker) are plotted (n = 3–4). Again, the standard deviations were high due to shifts in the gradient and have been removed to avoid a too disordered picture. The shift of fractions with the highest density of different markers also results in that the mean values don't reach 100% in most cases. The lines were added just to guide the eye.

We prepared a 10 000×g supernatant from rat brain and loaded this fraction on a discontinuous 2.5 to 30% iodixanol gradient. The γ-sectretase components nicastrin, PS1-CTF, PS2-CTF, Aph-1aL and Pen-2 were enriched in fraction 5–7 of this gradient, corresponding to an iodixanol concentration of 7.5–15% (Figure 1A), while the highest protein concentration was found in lighter fractions (Figure 1B). The direct substrate for γ-secretase cleavage, the APP-CTFs co-fractionated with the γ-secretase components whereas the full-length APP was more widely distributed (Figure 1A). To measure γ-secretase activity, the fractions were incubated at 37°C with or without the γ-secretase inhibitor L-685,458 and assayed for endogenous Aβ40 production by ELISA (Figure 1C). Since it is possible that the substrate concentration is a limiting factor, we also assayed for total γ-secretase activity by the addition of the exogenous substrate C99-FLAG which corresponds to β-secretase cleaved APP (Figure 1D). To calculate the γ-secretase dependent Aβ40 production, the Aβ40 levels found in the presence of the γ-secretase inhibitor L-685,458 were subtracted from the levels found in the absence of the L-685,458. Unfortunately, we were not able to detect Aβ42 in this experimental setup. Both the endogenous Aβ40 production and the total γ-secretase activity were enriched in fractions 5–7, although the peak activity fraction varied slightly between experiments. We quantified the levels of the subcellular markers by western blotting in the different fractions and found that the marker that showed the best correlation with γ-secretase activity was the early endosomal marker syntaxin 13 (Figure 1E). In addition, the plasma membrane marker N-cadherin correlated well with γ-secretase activity. The ER-Golgi intermediate compartment marker, ERGIC-53, and the *trans*-Golgi network marker γ-adaptin had two peaks of which one correlated with γ-secretase activity and the other one was found in lighter fractions. The ER marker KDEL was found in heavier fractions whereas the *cis*-Golgi marker GM130 was found in slightly lighter fractions although some overlap with γ-secretase occurred also for these markers (Figure 1E). Thus, active γ-secretase co-fractionates with an endosomal/plasma membrane enriched fraction in an iodixanol gradient prepared from rat brain.

### γ-Secretase Localize to Synaptic Membrane and Synaptic Vesicle Fractions

**Figure 2:**
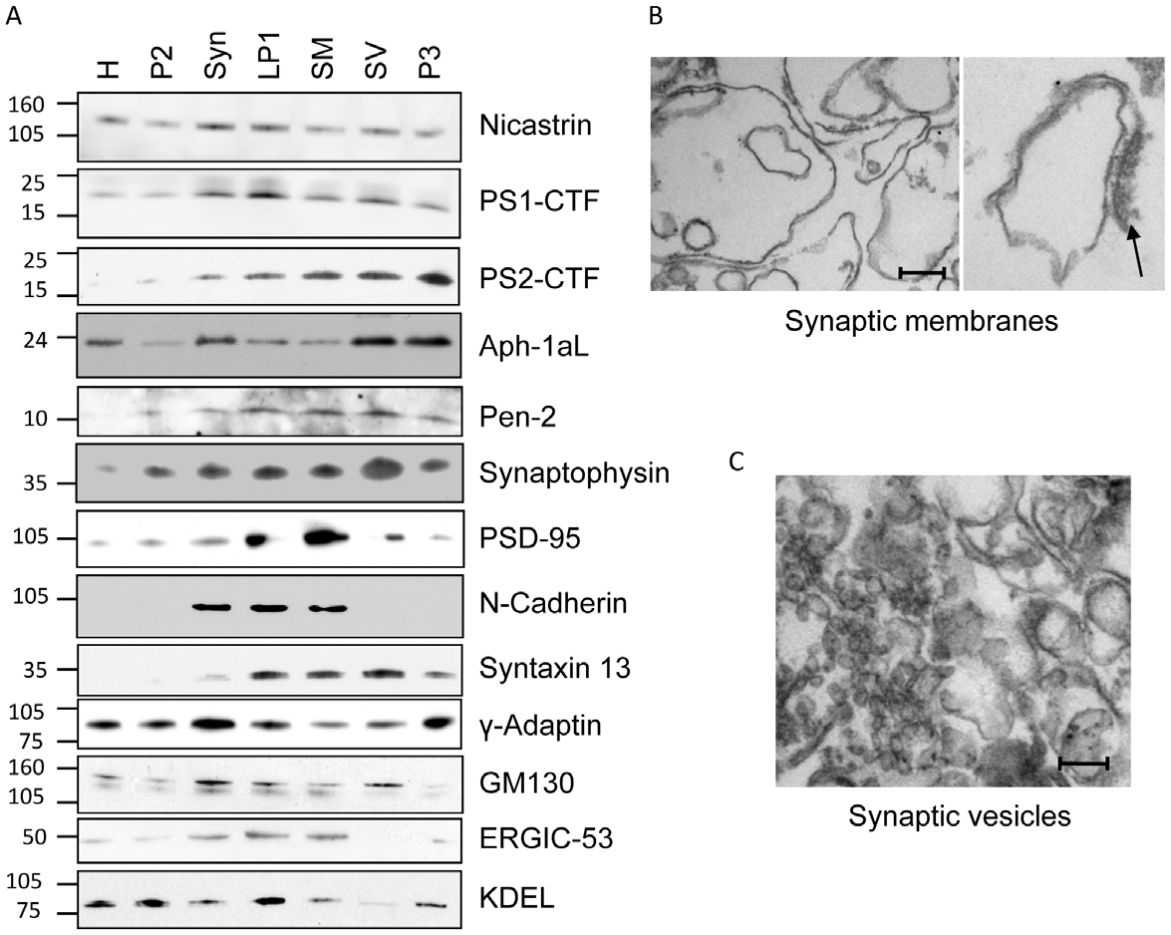
Preparation of synaptic membranes and synaptic vesicles from rat brain. A) Western blot for γ-secretase components and subcellular markers in the purification steps. synaptophysin (synaptic vesicles), PSD-95 (post-synaptic membrane), N-cadherin (plasma membrane), syntaxin13 (endosomes), γ-adaptin (trans-Golgi network), GM130 (*cis*-Golgi), ERGIC-53 (ER-Golgi intermediate compartment), KDEL (ER), H, homogenate; P2, 17 000×g pellet; Syn, Synaptosomes; LP1, lysed synapstosomes pellet, SM, synaptic membranes; SV, synaptic vesicles; P3, 100 000×g pellet from 17 000×g supernatant. Each marker was analyzed in 3–7 preparations. B) Electron micrographs of the synaptic membrane fraction showing structures resembling emptied synaptosomes with occasional attached post-synaptic densities (arrow). C) Electron micrograph of the synaptic vesicle fraction showing small vesicles of 40–50 nm but also some larger vesicles. Scale bar = 100 nm.

Since synaptic loss is one of the first pathological hallmarks of AD [25] and high local concentration of Aβ could induce this degeneration, we were interested in whether active γ-secretase was present at the synapse. In order to find out, we used a sucrose gradient protocol, followed by hypotonic lysis of synaptosomes to prepare synaptic membranes and synaptic vesicles [20]. The synaptic membrane markers PSD-95 and N-cadherin was enriched in the synaptic membrane fraction and of the synaptic vesicle marker synaptophysin was enriched in the synaptic vesicle fraction compared to the homogenate (Figure 2A). To get a quantitative measurement of synaptophysin and PSD-95, we loaded different amounts of homogenate, synaptic membranes and synaptic vesicles on a gel (data not shown) and came to the conclusion that synaptophysin was enriched 37±12 times (mean ± SD, n = 3) in the synaptic vesicle fraction compared to homogenate and PSD-95 was enriched 3 to 33 times in the synaptic membrane fraction depending on which gel-system that was used. The endosomal marker syntaxin 13 was also enriched in the synaptic membrane and synaptic vesicle fractions whereas the concentration of the *trans*-Golgi network marker γ-adaptin and the ER marker KDEL was decreased. In addition, the *cis*-Golgi-marker GM130 and the ER-Golgi intermediate compartment marker ERGIC-53 were sometimes found in the synaptic fractions but these apparent contaminations varied between experiments. Electron micrographs of the synaptic membrane fraction showed large membrane structures, probably representing emptied synaptosomes, with occasional attached post-synaptic densities (Figure 2B). The synaptic vesicle fraction indeed contained small vesicles of the expected size (∼50 nm) but also several larger vesicles, indicating that this was not a pure synaptic vesicle fraction (Figure 2C).The γ-secretase components were present in both the synaptic membrane and synaptic vesicle fractions, but the degree of enrichment varied between experiments.

**Figure 3:**
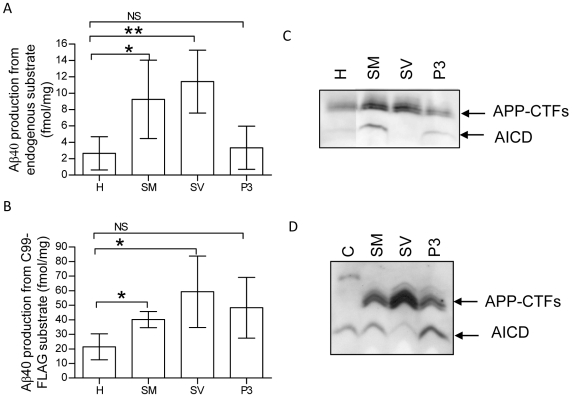
γ-Secretase activity in synaptic fractions. A) Aβ40 production from endogenous substrate was assessed by incubating the fractions at 37°C for 16 h with or without the γ-secretase inhibitor L-685,458. The Aβ40 levels were analyzed by ELISA and the levels in the samples with L-685,458 were subtracted from the levels without L-685,458. B) Aβ40 production after the addition of 20 ng of an exogenous substrate, C99-FLAG, was obtained as above with the difference that 20 ng of C99-FLAG was added prior to incubation. C) AICD production was assessed as above and the samples without L-685,458 were analyzed with immunoblotting. D) Degradation of AICD was investigated by adding synthetic AICD to the sample and incubating at 37°C for 16 h in the presence of L-685,458. H, homogenate; SM, synaptic membranes; SV, synaptic vesicles; P3, 100 000×g pellet; C = control (buffer and AICD only). Data are presented as mean values +/− SD (n = 4). *, p<0.05; **, p<0.01.

We found high Aβ40 production, assessed as above, from endogenous APP-derived substrates (Figure 3A) as well as from added C99-FLAG (Figure 3B) in both synaptic membranes and synaptic vesicles. The activity was clearly enriched as compared to the homogenate and the endogenous production was higher than in P3 (100 000×g pellet). P3 showed low Aβ40 production in this experiment but the AICD production was increased compared to homogenate (Figure 3C). Intriguingly, when we investigated the AICD production, we could not detect any AICD in synaptic vesicles whereas the AICD production in synaptic membranes was high (Figure 3C). There were no detectable AICD levels in the samples treated with L-685,458 (data not shown). To investigate whether the absence of AICD was due to AICD degradation, we incubated synthetic AICD with synaptic membranes, synaptic vesicles or the P3 pellet in presence of L-685,458. Whereas there was no degradation of AICD in synaptic membranes or in P3, the levels of AICD in synaptic vesicles was indeed dramatically decreased (Figure 3D). Regarding the intermediate purification steps, the production of both Aβ and AICD was low in the P2 and synaptosome fraction and similar to synaptic membranes in the LP1 fraction (data not shown).

**Figure 4:**
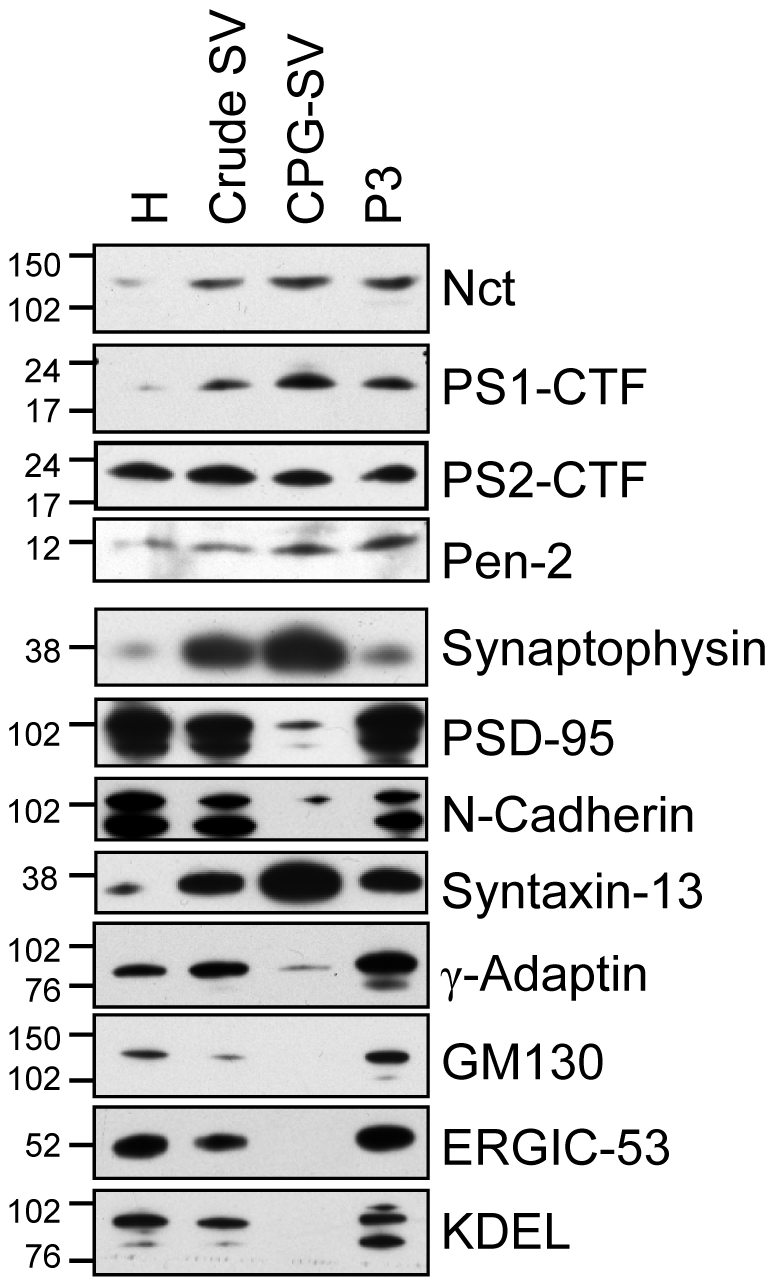
Presence of the γ-secretase components in a highly pure synaptic vesicle fraction. Highly pure synaptic vesicles were prepared using a protocol including controlled pored glass chromatography (CPG-SV) and analyzed for the presence of γ-secretase components and subcellular markers (see Figure 2), using Western blot. H, homogenate; Crude SV, synaptic vesicles used in Figure 2 & 3; P3, 100 000×g pellet.

Since the synaptic vesicle fractions analyzed above were quite crude, we investigated the presence of the γ-secretase components in synaptic vesicles purified using controlled pored glass chromatoghraphy (CPG). These vesicles were kindly provided by Dr. Matthew Holt, Göttingen, Germany and also this preparation contained high amounts of the γ-secretase components (Figure 4). The CPG-purified synaptic vesicles contained trace amounts of the trans-Golgi-network marker γ-adaptin and the plasma membrane markers N-cadherin and PSD-95 but no detectable levels of the Golgi and ER markers (Figure 4). The levels of the endosomal marker syntaxin-13 was still high but this is not necessarily due to the presence of endosomes since this marker has previously been detected in highly pure synaptic vesicles [26].

We conclude that both the synaptic membrane and the synaptic vesicle fractions have high γ-secretase activity and that the γ-secretase components are found also in highly pure synaptic vesicles.

### Confocal Microscopy Shows Co-Localization of γ-Secretase Active Sites with Endosomal, Synaptic and Golgi Markers

**Figure 5:**
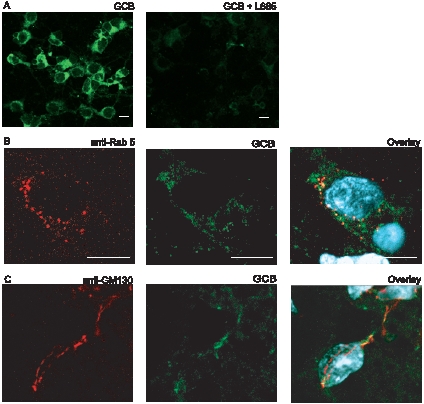
Labelling of γ-secretase active sites in rat brain sections. A) Labelling by GCB (γ-secretase inhibitor with a cleavable biotin moiety, green) is specific since it is competed out by a 100 x excess of the “cold” inhibitor L-685,458 (right panel). DAPI-staining (nucleus) in blue. B) Double labelling of the endosomal marker Rab5 (red) and GCB (green). C) Double labelling of the *cis*-Golgi marker GM130 (red) and GCB (green). Each experiment was performed 3 times. Scale bar = 10 µM.

**Figure 6:**
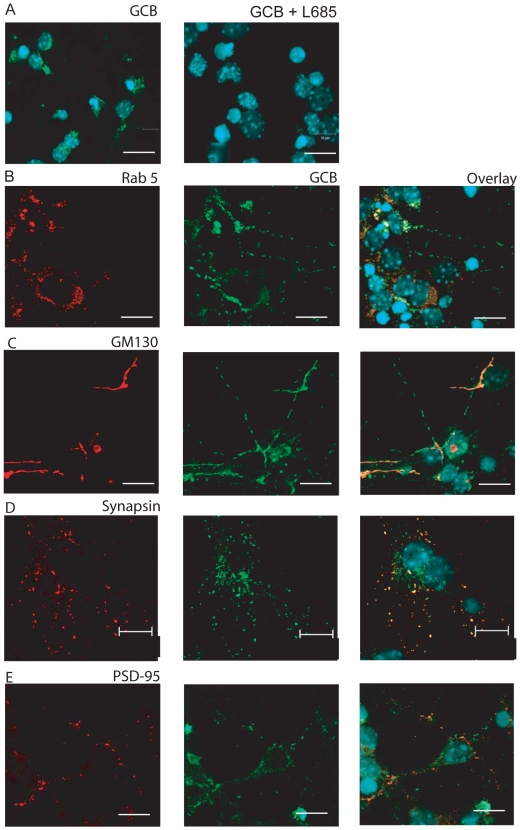
Labelling of γ-secretase active sites in mouse primary cortical neurons. A) Labelling by GCB (green) is specific since it is competed out by a 100 x excess of the “cold” inhibitor L-685,458 (right panel). DAPI-staining in blue. Double labelling of GCB (green) with B) the endosomal marker Rab5, C) the *cis*-Golgi marker GM130, D) the pre-synaptic marker synapsin and E) the post-synaptic marker PSD-95 (red). Each experiment was performed 3 times. Scale bar = 10 µM.

To determine the subcellular localization of γ-secretase active sites in intact brain sections, we labelled rat brain sections with a biotinylated derivative of the γ-secretase inhibitor L-685,458 (GCB, [24]) and streptavidin-Alexa488. The individual sections were also stained with antibodies for different subcellular markers. The binding of GCB was specific since it was competed by an excess of the unlabeled inhibitor L-685,458 (Figure 5A). In concordance with our results from the iodixanol gradient, we found partial co-localization of γ-secretase with the endosomal marker Rab5 in rat brain sections (Figure 5B). In addition, we detected some co-localization between active γ-secretase and the *cis*-Golgi marker GM130 (Figure 5C). We had difficulties finding conditions that were compatible with both GCB and synaptic markers in brain sections and thus we performed this double labeling on primary cortical neurons from mouse embryo. Also in this case the GCB labeling was competed by L-685,458 (Figure 6A). The neurons showed extensive co-localization of γ-secretase and the pre-synaptic marker synapsin (Figure 6D), whereas the co-localization with the post-synaptic marker PSD-95 was rare (Figure 6E). As in the case of rat brain, we found partial co-localization of active γ-secretase with both Rab5 (Figure 6B) and GM130 (Figure 6C) in the primary cortical neurons. In conclusion, confocal microscopy showed co-localization of active γ-secretase with endosomes, *cis*-Golgi and synaptic vesicles.

## Discussion

AD is a disease of the brain and therefore it is important to study the molecular mechanisms behind the disease in brain tissue. However, for one of the key players in the disease progress, γ-secretase, the number of studies performed in brain tissue is limited and the subcellular localization of this protease in brain remains elusive. For example, it is not known whether active γ-secretase is present at the synapse, since this was not examined in the earlier studies in non-neuronal cells. In this study, we used two approaches to determine the localization of active γ-secretase in rat brain. First, we used two different subcellular fractionation protocols; one using an iodixanol gradient and another one focusing on the preparation of synaptic fractions, and measured the γ-secretase activity in the fractions obtained. Second, we used a biotinylated γ-secretase inhibitor (GCB) to label γ-secretase active sites in rat brain sections. Both approaches indicated enrichment of active γ-secretase in endosomes and synaptic compartments.

Our results indicate endosomes as a major compartment for Aβ40 production in brain, since the endosomal marker syntaxin 13 was enriched in the same fractions as the γ-secretase activity in the iodixanol gradient. In addition, another endosomal marker, Rab5, partially co-localized with GCB-labelled γ-secretase in rat brain sections and primary cortical neurons. This is in agreement with earlier studies in cell lines, showing that endocytosis of APP is required for Aβ production [27], [28], [29], and that the γ-secretase components nicastrin, PS1 and Pen-2 co-fractionates with syntaxin 13 in a sucrose gradient [2]. Aβ produced in the endosomes can either remain in the endosomes [12], [13] or be secreted in exosomes via trafficking to multi-vesicular bodies [30].

We also found high γ-secretase activity in crude preparations of synaptic vesicles and synaptic membranes and these results were confirmed by the enrichment of the γ-secretase components in a highly pure synaptic vesicle preparation. In addition, confocal microscopy showed co-localization of the pre-synaptic marker synapsin and γ-secretase active sites in mouse embryonic cortical neurons. Furthermore, earlier studies have shown the localization of PS1 in different synaptic structures [14], [15], [17], [18]. Synaptic degeneration is an early event in AD and the pathological feature that correlate best with cognitive decline [25] and Aβ has been found to be synaptotoxic and to affect synaptic plasticity [31]. Our results suggest that the synaptotoxic Aβ is produced locally. γ-Secretase localized to the synapse could also have important physiological functions since conditional PS1/PS2 double deficient mice show impaired synaptic plasticity and memory followed by extensive neurodegeneration [32],

The endosomal and synaptic localization of active γ-secretase could be connected to each other since synaptic vesicle recycling is highly dependent on endocytosis [33] and it has recently been shown that increased Aβ production in response to increased synaptic activity is dependent on clathrin-mediated endocytosis [34]. We found the endosomal marker syntaxin 13 also in our highly pure synaptic vesicle preparations indicating either that also this preparation was contaminated by endosomes or that this marker are present in synaptic vesicles. In favour of the latter hypothesis, this marker were found in synaptic vesicle preparations where the purity was confirmed using electron microscopy [26]. These data together with our histochemical labelling of γ-secretase active sites, that indicates a pre-synaptic rather than post-synaptic localization in primary embryonic mouse neurons, strongly suggest synaptic vesicle and/or pre-synaptic endosomes as a main subcellular localization of γ-secretase.

Along with the finding of high γ-secretase activity in synaptic membranes, the plasma membrane marker N-cadherin also correlated well with activity in the iodixanol gradient. Labelling of active γ-secretase in an approach similar to ours has demonstrated the existence of γ-secretase on the cell surface in cell culture [35], and Chyung *et al* [9] showed that around 6% of the total γ-secretase is present at the cell surface.

In addition to endosomes and synaptic structures, we detected sparse co-localization of the *cis*-Golgi marker GM130 with the γ-secretase in rat brain sections and this co-localization was more pronounced in mouse primary neurons. In accordance with this, we found some γ-secretase activity in the Golgi-enriched fractions in the iodixanol gradient although the peak fractions of γ-secretase activity did not correlate with GM130 expression and high γ-secretase activity in Golgi/*trans*-Golgi network has earlier been observed in different cell lines [3], [4].

It is possible that other subcellular compartments than the ones mentioned above contribute to the γ-secretase activity since some other markers overlapped with γ-secretase activity in the iodixanol gradient and the synaptic fractions contained traces of non-synaptic markers. In addition, the GCB labelling shows that all γ-secretase cannot be attributed to a single subcellular compartment. In line with this, it has been shown that different substrates could be processed at different subcellular locations. For example, whereas most APP processing occurs inside the cells, Notch processing mainly occurs at the cell-surface [36].

We also investigated the levels of the different γ-secretase components in the different fractions. In the iodixanol gradient the components correlated well with γ-secretase activity. In contrast, although the γ-secretase activity was highly enriched in the synaptic fractions, the enrichment of the components was less pronounced. The high activity is not solely explained by the enrichment of the substrate levels (APP CTFs) since the γ-secretase activity was enriched also when an exogenous substrate was added to the reaction. One explanation could be that the γ-secretase components only partially are assembled into an active complex and that the individual components are more widely distributed.

Interestingly, the AICD levels were very low in the synaptic vesicle fraction despite high Aβ levels. This was probably due to degradation since synthetic AICD was degraded to a higher degree in synaptic vesicles than in other fractions. Insulin degrading enzyme (IDE) is the main candidate for AICD degradation [37], and it can also degrade Aβ [38] but no Aβ degradation was observed in the synaptic vesicle fractions. However, AICD and Aβ are released to the opposite sides of the membrane, and it is possible that IDE in this case is compartmentalized in such a way that it only degrades AICD. Alternatively, there might be other AICD degrading enzymes that are enriched in synaptic vesicles.

In summary, our study indicates endosomes and/or synaptic structures as the main compartments of active γ-secretase in brain, and that other subcellular compartments contribute to a minor degree. The knowledge about the subcellular localization of Aβ40 production may help to develop drugs that intervene with this process. In addition, the fractions found to be enriched in γ-secretase activity can be used for further studies requiring highly active γ-secretase.
